# Mirror Illusion Modulates M1 Activities and Functional Connectivity Patterns of Perceptual–Attention Circuits During Bimanual Movements: A Magnetoencephalography Study

**DOI:** 10.3389/fnins.2019.01363

**Published:** 2020-01-14

**Authors:** Chia-Hsiung Cheng, Szu-Hung Lin, Ching-Yi Wu, Yi-Han Liao, Ku-Chou Chang, Yu-Wei Hsieh

**Affiliations:** ^1^Department of Occupational Therapy, Graduate Institute of Behavioral Sciences, Chang Gung University, Taoyuan City, Taiwan; ^2^Laboratory of Brain Imaging and Neural Dynamics (BIND Lab), Chang Gung University, Taoyuan City, Taiwan; ^3^Healthy Aging Research Center, Chang Gung University, Taoyuan City, Taiwan; ^4^Department of Psychiatry, Chang Gung Memorial Hospital, Linkou, Taiwan; ^5^Department of Physical Medicine and Rehabilitation, Chang Gung Memorial Hospital, Linkou, Taiwan; ^6^Division of Cerebrovascular Diseases, Department of Neurology, Kaohsiung Chang Gung Memorial Hospital, Kaohsiung City, Taiwan; ^7^College of Medicine, Chang Gung University, Taoyuan City, Taiwan; ^8^Discharge Planning Service Center, Kaohsiung Chang Gung Memorial Hospital, Kaohsiung City, Taiwan; ^9^Department of Senior Citizen Service Management, Yuh-Ing Junior College, Kaohsiung City, Taiwan

**Keywords:** visual conflicts, motor cortex, beta rebound oscillation, functional connectivity, magnetoencephalography

## Abstract

We differentiated the influence of mirror-induced visual conflicts on the perceptual–attention–motor control process by examining the variation of primary motor cortex (M1) activities and the functional connectivity among five brain regions associated with perceptual, motor, and attentional processes. Magnetoencephalography (MEG) was recorded under three conditions: both hands kept stationary with the forearms supinated (resting condition), in-phase bimanual movements with congruent visual feedback [symmetry (Sym) condition], and out-of-phase bimanual movements with incongruent visual feedback [asymmetry (Asy) condition]. We found that compared with the resting state, the decrease in beta oscillation was greater in the Sym than in the Asy condition, suggesting a greater activation of M1 when implementing hand movement without visual conflict. The results of functional connectivity patterns showed that the alpha band functional connectivity between V1 and superior temporal gyrus (STG) and the gamma band functional connectivity between the precuneus and posterior cingulate cortex (PCC) triggered greater or slightly greater coherence strength in the Asy condition than in the Sym condition. However, the beta band functional connectivity showed no difference between the two conditions in all pairs of the brain regions. These findings confirm and extend the previous findings to provide evidence that mirror visual feedback engages the functional networks associated with the perceptual–attentional process and triggers M1 activation, although the M1 activation is functionally independent of other brain regions unrelated to motor function. In summary, this study demonstrated a concrete functional connectivity pattern for motor control in the face of visual conflicts, and providing a foundation for future research to examine the dynamic functional networks of mirror illusion in motor control.

## Introduction

Delicate motor control relies on the integration of multiple sensory feedbacks to support the performance of ongoing acts, such as integrating the somatosensory feedbacks from peripheral receptors, and the visual cues associated with the positions of body parts (for a review, see Scott, 2004). The importance of sensory feedbacks to motor control has been evident from numerous kinematic studies (e.g., Connolly and Goodale, 1999; Saunders and Knill, 2003). These studies used mirror-induced visual feedback (MVF) to investigate how continuous online MVF of a moving hand contributed to motor control throughout the whole course of bimanual reaching movement. The findings suggested that MVF could facilitate movement control through the imitating connections between visual inputs and motor/premotor areas (Altschuler et al., 1999). The rehabilitation of patients with stroke also leverages on the advantage of MVF and employed mirror therapy to improve the control of the affected upper limb (for a review, see Deconinck et al., 2015).

Previous magnetic resonance imaging (MRI) studies suggested that MVF modulates motor cortical activations and that such motor activation might be mediated in a different pathway, such as perceptual and attentional pathways. For example, a functional MRI (fMRI) study found that the superior temporal gyrus (STG) and superior occipital gyrus, rather than motor or premotor areas, were activated by the MVF of hand movements (Matthys et al., 2009). Furthermore, MVF could trigger neural activities of the precuneus and posterior cingulate cortex (PCC) during bimanual movements (Michielsen et al., 2011). The precuneus and posterior parietal cortex have also been reported to be associated with visual-motor coordination (Fink et al., 1999; Michielsen et al., 2011) and spatial attention (Moffat, 2009; Bakola et al., 2010), which is demanding during the MVF. In this case, the modulations of MVF on the primary motor cortex (M1) might be mediated through different pathways or as a form of network. Clinical reports on MVF in stroke patients have attributed the benefits of motor function to the activation of the mirror neuron system (MNS), which consists of M1, the inferior parietal lobe, inferior frontal gyrus, and STG (Ezendam et al., 2009; Ramachandran and Altschuler, 2009; Rizzolatti et al., 2009).

Although several studies have focused on how MVF modulates cortical activation, these findings did not reach a definite conclusion on the brain modulation (or adaptation) and did not reveal how M1 works with other brain areas and temporal association among brain regions. Furthermore, no studies to date have examined the comprehensive functional connectivity of the perceptual, attentional, and motor network in the situation of MVF conflict.

Instead of the nature of MRI specializing in high spatial resolution, magnetoencephalography (MEG) takes advantage of excellent temporal resolutions and reasonable spatial resolutions. Moreover, a distributed source modeling method (minimum norm estimate) used in MEG studies has made MEG a suitable device for clarifying the changes of regional activation and network connectivity at the source level (Lin et al., 2006a, b). Very few studies have used MEG to underscore the mechanism of MVF (for a review, see Deconinck et al., 2015; Zhang et al., 2018). One seminal study by Butorina et al. (2014) investigated the association between visual inputs and activation of the sensorimotor cortex and showed that MVF from a moving hand induced high gamma oscillation (55–85 Hz) response in the sensorimotor cortex of both hemispheres. To further study the effects of MVF on the motor cortical activation, the electricity-induced beta (∼20 Hz) rebound oscillation can be applied to monitor the changes of motor cortical activation including M1. Beta rebound oscillation, which peaks approximately 400 to 900 ms after the median nerve stimulation, is generated in the M1 (Pfurtscheller et al., 1996; Cassim et al., 2001; Gaetz and Cheyne, 2006). This rhythmic activity is clearly observed when the subject’s hand is at the resting position but is decreased during the observation of hand movements and abolished during voluntary movements (Hari et al., 1998; Järveläinen et al., 2004; Ichikawa et al., 2007; Cheng et al., 2017a). Thus, this rhythmic activity is consistently considered as an indicator of the M1’s functional status. Our previous MEG study, for example, demonstrated that beta rebound oscillation was less suppressed in the observation of abnormal hand movements than normal ones, suggesting a weaker M1 activation during observing awkward, distorted movement patterns (Cheng et al., 2017a).

This study had two aims. First, we examined the effects of mirror-induced visual conflicts on M1 activities, with the beta rebound oscillation as an indicator. Second, we investigated how the visual conflicts modulate the functional connectivity among the brain areas associated with perceptual, motor, and attentional processes. Specifically, we classified the oscillatory activities of these coherences and averaged them into alpha, beta, and gamma bands that had been verified as indicators related to perceptual, motor, and attentional process (for a review, see Deconinck et al., 2015). To achieve these two aims, we conducted an experiment in which the visual feedback was congruent or incongruent with the motor execution in addition to a control condition (resting condition). A congruent MVF was that the participants performed the in-phase bimanual movements using a mirror box in the middle of two hands that covered their left non-dominant hands and reflected the mirror image of their right hands. The incongruent MVF was the same setup while the participants performed the out-of-phase bimanual movements.

## Materials and Methods

### Participants

The study participants were 19 right-handed healthy volunteers (10 women). Data from 4 subjects were discarded due to substantial artifacts or other technical problems. The mean age of the 15 remaining subjects (8 women) was 22.73 ± 0.41 years. All participants provided written informed consent approved by the Institutional Review Board of Taipei Veterans General Hospital (Taipei, Taiwan).

### Stimulation

During the entire experiment, the left median nerve was stimulated at the wrist with 0.2-ms square-wave pulses by a saddle-type electrode. The interstimulus interval varied from 1.8 to 2.2 s to avoid expectation effects. Stimulus intensity was 1.2 times the motor threshold of the abductor pollicis brevis to obtain the cortical responses with a better signal-to-noise ratio (Cheng et al., 2017a, b, 2018). Subjects were instructed to ignore the stimulation and to focus on the tasks we requested. We collected at least 100 artifact-free trials in each condition (see below) for further analyses.

### Experimental Procedures

**FIGURE 1:**
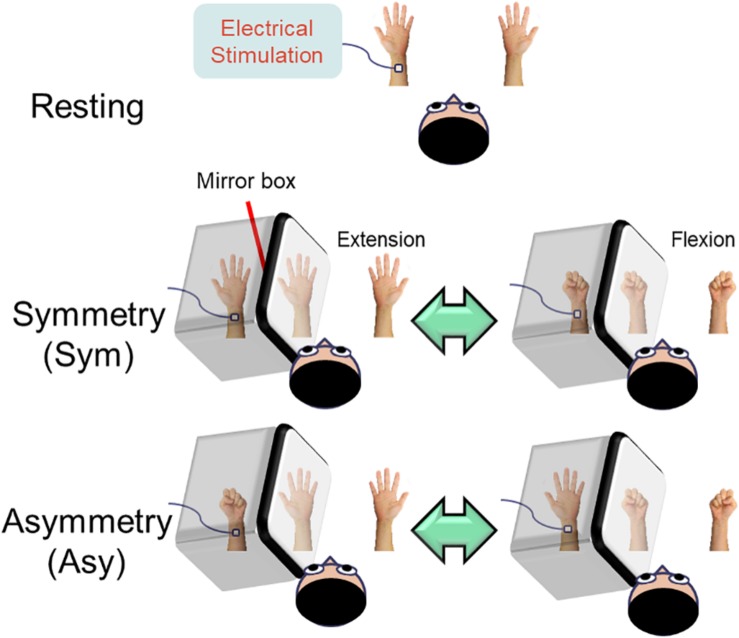
Illustration of the experimental design. In the resting condition, the subjects were instructed to look at both stationary hands. In the symmetry (Sym) condition, the subjects performed bimanual in-phase fingers flexion/extension with a mirror covering the left hand. In the asymmetry (Asy) condition, the subjects performed bimanual out-of-phase fingers flexion/extension with a mirror covering the left hand, which produced a conflict between hand movements and visual feedback. During the whole experimental procedure, the left median nerve was stimulated at the wrist in order to probe the beta rebound activities of the motor cortex.

Participants comfortably sat upright with their heads resting in the helmet-shaped MEG device (Vectorview, Elekta-Neuromag, Helsinki, Finland). Neuromagnetic responses were recorded in three conditions in a randomized order (Figure 1).

#### Resting

Both hands were kept stationary with the forearms supinated. The subjects were instructed to look at both stationary hands.

#### Symmetry (Sym)

Participants’ left hand was precisely covered by a mirror so that the mirror reflection of the right hand overlapped the view of the masked left hand. In this condition, participants were instructed to perform bimanual in-phase fingers flexion/extension repetitively with a frequency of approximately 1 Hz to receive a congruent MVF.

#### Asymmetry (Asy)

All of the settings were similar to the symmetry (Sym) condition except that participants were instructed to perform bimanual out-of-phase fingers flexion/extension repetitively with a frequency of approximately 1 Hz to receive an incongruent MVF.

Before the MEG recordings, substantial practice of 3 to 5 min was delivered to each participant to ensure that they were very familiar with in-phase and out-of-phase hand movements with a constant frequency at approximately 1 Hz.

### Magnetoencephalography Recordings

Neuromagnetic activities were recorded using a 306-channel Neuromag system at Taipei Veterans General Hospital. MEG data were sampled at 1000 Hz with an online bandpass filter of [0.1, 200] Hz. The head position relative to the MEG sensors was registered at the beginning of each block by measuring the magnetic signals produced by current leads to four head position indicators at the forehead (left and right) and bilateral mastoids. The three fiducial points based on Cartesian coordinate system were determined using a three-dimensional digitizer. The *x*-axis ran from left to right preauricular points and the *y*-axis passed through nasion, and the *z*-axis pointed in an inferior–superior direction.

The MEG data were processed with Maxfilter software based on the temporal extension of the signal space separation algorithm to reduce artifacts originating inside and outside the MEG (Taulu et al., 2004).

### Analysis of M1 Beta Rebound Oscillation

The modeling of cortical responses was implemented in Brainstorm software (Tadel et al., 2011). At the beginning, the artifacts contaminated by eye blinks were removed using signal space separation. The forward problem of MEG measures was resolved by means of the overlapping-sphere method (Huang et al., 1999). The depth-weighted minimum norm estimate was used to compute cortically constraint source activation, with over ∼7500 elementary dipole locations in each hemisphere. The individual source maps were geometrically rescaled to the ICMB 152 brain template by Brainstorm’s registration methods.

To calculate the power spectrum, we removed evoked response from each trial of the raw data (100 ms before and 1000 ms after the stimulus onset), and then the data in the identified regions of interest (ROIs) were transferred using Morlet-wavelet time–frequency decomposition, with a central frequency of 1 Hz and a time resolution of 3 s. The power of signal fluctuations was estimated and exhibited between 1 and 50 Hz in 1-Hz steps.

The mean strength of the most reactive beta oscillation (2 Hz for consecutive bins) in M1, approximately 4 to 5 cm^2^, was identified and calculated from the average of 200 ms centering peak latency of beta rebound activities (i.e., 100 ms before and 100 ms after the peak) (Cheng et al., 2016, 2017a,b, 2018). The time-resolved magnitude of each elementary dipole was normalized to its fluctuations over the pre-stimulus baseline, yielding a set of *z*-score time series. We calculated the mean power of the beta rebound oscillation in each condition from all of the participants.

The suppression index of beta power suppression in the Sym and Asy conditions with respect to the resting condition was used to indicate the magnitude of M1 activation: [suppression index = [(β_resting_ – β_Sym or Asy_)/β_resting_] × 100%]. Hence, a suppression index with a larger value suggested higher M1 activation.

### Analysis of Functional Connectivity

**FIGURE 2:**
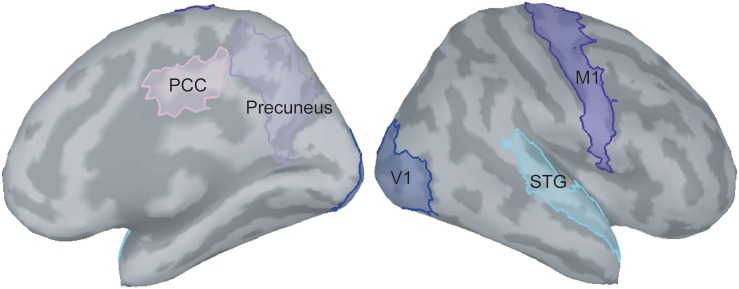
Selection of regions of interest on the ICBM152 cortical surface. PCC, posterior cingulate cortex; V1, primary visual cortex; M1, primary motor cortex; STG, superior temporal gyrus.

In the present study, we were particularly interested in how the visual feedback modulates M1 activities; therefore, the ROIs situated in the pathway between V1 and M1 were selected. According to the previous literature (Matthys et al., 2009; Michielsen et al., 2011; Butorina et al., 2014), we identified the V1, M1, precuneus, PCC, and STG as the ROIs to perform functional connectivity analysis (Figure 2). The procedures of time–frequency decomposition were the same as those aforementioned. The entire epoch (i.e., 1000 ms) of each raw trial was used to compute functional connectivity. The source-based coherence among the V1, M1, precuneus, PCC, and STG was estimated by using magnitude-squared measures, with maximum frequency resolution of 1 Hz and highest frequency of interest of 50 Hz. Oscillatory activities of these coherence measures were classified and averaged into alpha (8 to 12 Hz), beta (13 to 30 Hz), and gamma (31 to 50 Hz) bands.

### Statistical Analysis

The data are presented as mean ± standard error of the mean. To avoid increasing vulnerability to assumption violations of parametric analysis and type I errors, the suppression index of beta power and functional connectivity differences between Sym and Asy conditions were evaluated by non-parametric Wilcoxon sign rank tests with Monte Carlo simulation for estimating *p* values (one-tailed). The critical *p* value of statistical significance was set at 0.05. For the functional connectivity, we infer our results based on three frequency bands of 10 comparisons (10 pairs of brain regions). Therefore, we used the Benjamini–Hochberg procedure (Benjamini and Hochberg, 1995) to adjust the *p* values of 10 comparisons on each frequency band.

## Results

**FIGURE 3:**
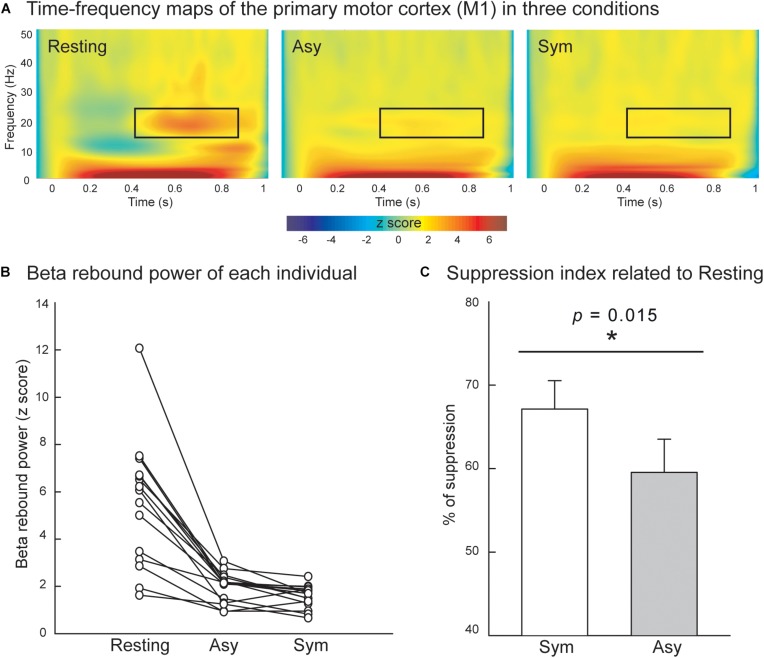
**(A)** Grand-averaged time–frequency maps of electricity-induced beta rebound oscillations (black rectangles) of the right primary motor cortex (M1) in the resting, Asy, and Sym conditions. **(B)** The M1 beta rebound strength from each individual in the resting, Asy, and Sym conditions. **(C)** The suppression index refers to the extent of beta power suppression in Sym and Asy with respect to the resting condition. The beta rebound oscillations were suppressed more in the Sym than in the Asy condition. ^∗^Represents significant effect (*p* < 0.05).

Figure 3A displays the grand-averaged time–frequency maps over the time interval of −100 to 1000 ms and the frequency band from 1 to 50 Hz in the right M1. The electricity-induced beta oscillation was decreased immediately after the left median nerve stimulation and then rebounded above the pre-stimulus baseline level at the time window of 400 to 900 ms when the subjects were in the resting condition. We identified the peak latency of beta rebound activities within this time window (400 to 900 ms, *M* = 685.5 ms, *SD* = 140.7 ms, the shortest = 500 ms, the longest = 900 ms) from all of the participants and calculated from the average of 200 ms centering peak latency of beta rebound activities (i.e., 100 ms before and 100 ms after the peak) to indicate the mean power of the beta rebound oscillation in each condition. The mean power of the beta oscillation was substantially reduced in the Asy and Sym conditions compared to the resting condition, as also shown in Figure 3B. The statistical results of suppression index further demonstrated that beta oscillation was suppressed much more in the Sym condition than in the Asy condition (*Z* = −2.17, *p* = 0.015), suggesting that M1 would be more activated when hand movements were congruent with the visual feedback images (Figure 3C).

**FIGURE 4:**
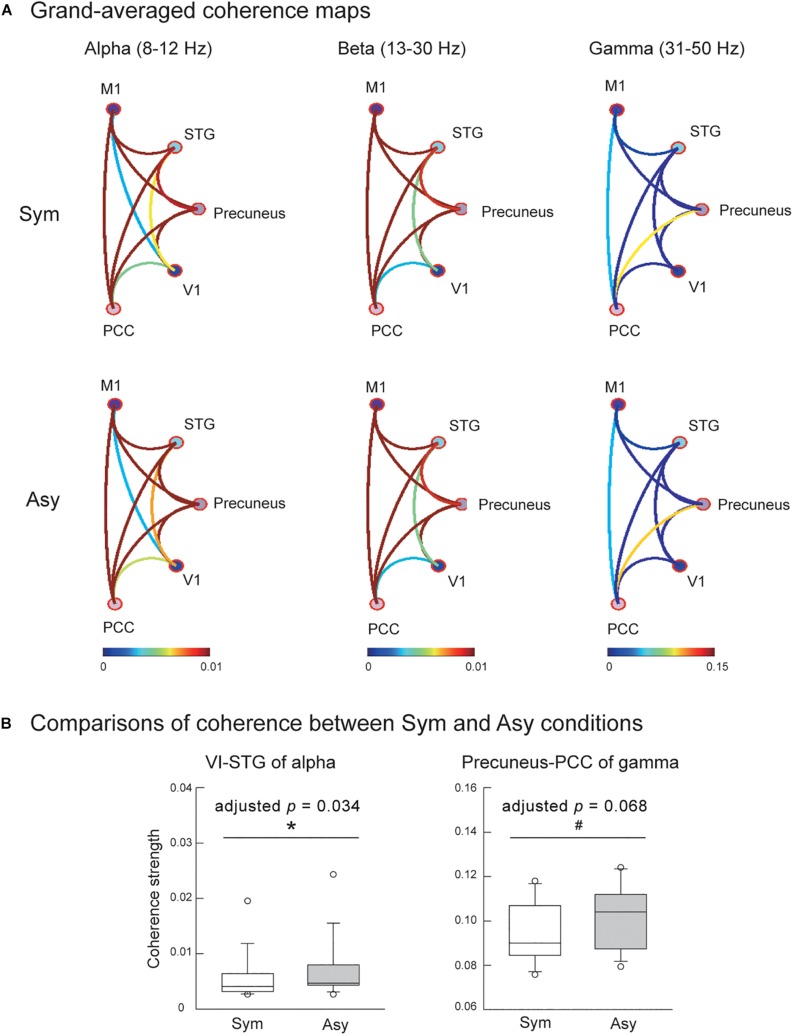
**(A)** Grand-averaged functional connectivity among primary motor cortex (M1), superior temporal gyrus (STG), precuneus, primary visual cortex (V1), and posterior cingulate cortex (PCC) in the Sym and Asy conditions. Different colors of connectivity lines representing varying degrees of coherence strength. **(B)** The statistical results showed that functional connectivity of V1-STG (alpha band) was stronger in the Asy than in the Sym condition, and the functional connectivity of precuneus-PCC (gamma band) demonstrated a trend to be significantly stronger in the Asy than in the Sym condition. ^∗^Represents significant effect (*p* < 0.05); ^#^represents marginal effect (*p* < 0.01).

Also evaluated was the effect of visual conflicts on the functional connectivity among the V1, M1, precuneus, PCC, and STG. Figure 4A displays the connectivity maps across all of the participants in the Sym and Asy conditions for the alpha, beta, and gamma bands. Statistical results demonstrated that the cortical coherence of the alpha bands between V1 and STG (V1-STG) was significantly stronger in the Asy condition (*Z* = −2.56, *p* = 0.003, adjusted *p* = 0.034). The cortical coherence of gamma bands between the precuneus and PCC (precuneus-PCC) showed marginally significant difference (*Z* = −2.42, *p* = 0.007, adjusted *p* = 0.068), suggesting a stronger functional connectivity in the Asy condition. No other significant or marginal effects were found (adjusted *p*s > 0.27) (Figure 4B).

## Discussion

This study aimed to differentiate the influence of two types of mirror visual feedback (MVF) on the perceptual–attention–motor control processes by examining the variation of M1 activities and the functional connectivity among five brain regions (V1, STG, precuneus, PCC, and M1). The results of beta power oscillation for M1 indicated that the M1 ∼20-Hz activity was reduced compared with the resting state when participants received an MVF of their uncovered hand’s movement in a symmetric and asymmetric manner. Specifically, the decrease in beta oscillation was greater in the Sym condition than in the asymmetry (Asy) condition, suggesting a stronger activation of M1 when implementing hand movement without visual conflict. Regarding the functional connections between five specified brain regions, we found that the alpha band functional connectivity between V1 and STG and the gamma band functional connectivity between precuneus and PCC showed greater or slightly greater coherence strength under asymmetric MVF than symmetric MVF. These findings indicated that MVF engages functional networks of the brain regions related to the perceptual process (Matthys et al., 2009; Doesburg et al., 2016) and may also be dedicated to the attentional process (Cavanna and Trimble, 2006; Leech et al., 2012), while the previous studies focused only on one or part of them.

### Beta Rebound Oscillation in M1

Consistent with the previous findings (Hari et al., 1998; Järveläinen et al., 2004; Ichikawa et al., 2007; Cheng et al., 2017a), we found that the beta oscillations in M1 were reduced under Sym and Asy compared with the resting state. In addition, greater beta power oscillations were shown in the Asy condition rather than in the Sym condition, confirming the findings of our previous MEG study (Cheng et al., 2017a); that is, beta rebound oscillation was less suppressed in the observation of abnormal hand or grasping-reaching movements than in normal ones. Asy movement led to reduced M1 activity, extending the findings that a weaker M1 activation occurs not only during observing awkward, distorted movement patterns but also when out-of-phase bimanual tasks are performed.

### Functional Connectivity Between Brain Regions Associated With Perceptual Process

The coherence strength of alpha band connectivity between V1 and STG was significantly increased under asymmetric MVF compared with symmetric MVF, indicating that MVF is required to activate a network dedicated to visual perception and motion imagery for processing and performing an asymmetric MVF task. The V1 was characterized as directly interconnected with posterior extrastriate areas V2, V3, V3A, V4, and MT (Felleman and Van Essen, 1991), and evidence from numerous neurophysiological and neuroimaging studies suggested that these recurrent loops between V1 and posterior extrastriate areas were essential to maintain a visual representation in consciousness (for a review, see Tong, 2003). Moreover, the STG was often linked to its neighboring superior temporal sulcus and was considered to be associated with the MNS (Matthys et al., 2009). The function of this region was involved in the visual identification of biological motion (Schultz et al., 2004). Evidence from previous studies considering early blindness confirmed that V1 and STG were functionally connected (Yu et al., 2007; Burton et al., 2014). The increase in the coherence strength of V1 and STG under asymmetric MVF could result from the increased activation of both the visual area and the MNS. The participants possibly make additional efforts to retain the visual illusion from mirror feedback and process the visualized movement in the MNS for subsequent asymmetric movement performance.

The results of the effect of MVF on the alpha band functional connectivity between V1 and STG were consistent with the perspective that alpha oscillations (8–12 Hz) were considered to be a local marker of the somatosensory and visual cortices excitability level, with a smaller alpha power being associated with greater excitability (Pfurtscheller and Lopes da Silva, 1999; Anderson and Ding, 2011; Lebar et al., 2017). Lebar et al. (2017), for example, found that alpha power significantly decreased in the visual cortex, indicating an increased gain of visual inputs during sensory incongruence compared with unperturbed conditions. Thus, this study further confirmed the role of alpha oscillation on visual sensory detectability and discriminability.

However, the functional connectivity between V1 and other brain regions (PCC and precuneus) associated with attentional process of both types of MVF showed similar coherence strengths. The significant difference between both types of MVF only occurred in the visual–perceptual connectivity but not in the visual-attention connectivity, indicating the MVF conflict mainly recruited the visual–spatial information process to differentiate the different phase of both hands and did not require attention, possibly because both hands still shared the same movement patterns in both conditions.

These findings are inconsistent with the results of the study by Doesburg et al. (2016). One possibility is that we did not consider the function roles in the subdivisions of the ROI of each brain region we chose. Three distinct patterns of functional connectivity are present within the precuneus (Margulies et al., 2009). The posterior precuneus is functionally connected to the visual cortical regions, the central part is connected to the dorsolateral prefrontal cortex, and the anterior part has connections with medial somatomotor regions. Not considering the individual subdivisions might obscure the potential connections of certain subdivisions of precuneus with V1. Considering separate functional subdivisions within each brain region is necessary when examining the underlying mechanism of the MVF effects on the motor control.

### Functional Connectivity Between Brain Regions Associated With Attentional Process

Two types of MVF showed a marginally significant effect (adjusted *p* = 0.068) of varying coherence strength of the functional connectivity between the precuneus and PCC in gamma band (31–50 Hz), which might suggest that MVF could activate a network dedicated to attention and action monitoring. The PCC, highly interconnected with various brain regions, is considered a hub for information exchange (Hagmann et al., 2008) and has a prominent role in the cognitive control of behavior (Leech et al., 2012). The nearby precuneus was known to be involved in processing visuospatial information and directing spatial attention, especially during bimanual coordination tasks (Wenderoth et al., 2005). Notably, a number of studies had shown that the precuneus was particularly active during self-centered mental imagery strategies (Cavanna and Trimble, 2006), which could account for the combined effect of imagery and MVF. Thus, marginally greater coherence strength under asymmetric MVF than symmetric MVF might imply that greater attentional demand was required to resolve the conflict between expected and actual visual feedback to monitor the task being successfully continued. However, this is only the marginal effect we found in this study, and further examinations are required in future studies to verify the role of the attentional process in the effect of mirror-induced visual conflicts.

### Functional Connectivity Between Brain Regions With Beta Band

Oscillations in the beta frequency band (13–30 Hz) are known to be important in movement, and previous studies have suggested that the ∼20-Hz activity was present at rest and was suppressed during movement in M1 (Pfurtscheller and Lopes da Silva, 1999; Tominaga et al., 2009; Muthukumaraswamy et al., 2013). However, we did not find any significant effect of the beta band functional connectivity between the M1 and other brain regions. This finding might indicate that the MVF could engage the functional networks associated with the perceptual–attentional circuits and triggers M1 activation, and the M1 activation is functionally independent of with other brain regions. To the best of our knowledge, no studies have directly examined the beta band functional connectivity of M1 and other brain regions during mirror-induced visual conflicts, and further research is required to further explore the effects of MVF on functional connectivity between M1 and other brain functional regions.

### Study Limitations and Directions for Future Research

One might argue that this study only selected half hemispheric ROIs and its Asy between two hemispheres, particularly given that both hands were moved. However, one of our purposes of this study was to investigate how the visual conflicts modulate the functional connectivity among the brain areas associated with perceptual, motor, and attentional processes, so we selected the ROIs based on previous research that had suggested these brain areas were involved in these functional processes. We only select ROIs in the right hemisphere because the MVF conflict was only induced by the left-hand side, and we were interested in how this conflict induced functional connectivity among corresponding brain areas. Furthermore, one might not be convinced with the interpretation of the functional network results because of the lack of behavioral results to go with brain response for the corresponding functional processes. Evidence from numerous neurophysiological and neuroimaging studies have demonstrated the function roles of our identified ROIs (Schultz et al., 2004; Leech et al., 2012; Burton et al., 2014; for a review, see Tong, 2003; Wenderoth et al., 2005; Yu et al., 2007). Thus, although the present study lacks behavioral measures associated with brain responses, it is appropriate to infer our imaging results through the functional processes correspondingly based on previous evidence.

We examined a correlation within and between three functional networks (attentional process, MNS, and motion process) across five brain regions (V1, precuneus, PCC, STG, and M1), but the causal relations between these functional networks remain uncertain. Hence, future studies that use non-invasive brain stimulation to selectively target these functional networks to investigate causal relationships among these functional networks are encouraged. Furthermore, we did not consider the functional roles of each brain region’s subdivisions, just as demonstrated by Margulies et al. (2009). The whole picture of functional networks associated with the perceptual–motor process deserves more research attention. In the future, individual functional subdivisions within each brain region should be considered when examining the underlying mechanism of the MVF effects on the motor control. To further examine the possible mechanism of MVF used in mirror therapy for stroke rehabilitation of upper limb, future research may recruit patients with upper-limb disability, or require normal participants to imitate patient’s paretic upper extremity movement (e.g., partial-ranged fingers flexion/extension).

## Conclusion

To our knowledge, the present study is the first to investigate effects of MVF on the functional connectivity among brain regions associated with perceptual–attention–motor control processes. In addition, to demonstrate M1 activation patterns during two types of MVF conditions, we confirm that MVF activates the functional networks dedicated to perceptual (V1 and STG) process and may also dedicate to the attentional (precuneus and PCC) process, and the M1 activation is functionally independent of other brain regions. These findings are consistent with known motor learning principles that attribute success of motor implementation to visual–spatial perceptual focus and attentional processing.

In summary, although further research is warranted to fully understand the potential of MVF in motor processing, this study still provides a plausible functional connectivity pattern for this process, with different types of functional networks triggering activities of their respective frequency bands. This pattern provides a foundation for future research to examine the dynamic functional networks among the distributed brain regions in the face of visual conflicts.

## Data Availability Statement

The datasets for this manuscript are not publicly available because this research has been supported by many funding foundations, and we are unable to determine the availability of data at our discretion. Requests to access the datasets should be directed to cywu@mail.cgu.edu.tw.

## Ethics Statement

All participants provided written informed consent approved by the Institutional Review Board of Taipei Veterans General Hospital (Taipei, Taiwan).

## Author Contributions

C-YW, C-HC, and S-HL contributed to the conception of the study. C-YW and C-HC contributed to the methodological design. Y-HL contributed to the data acquisition. C-HC organized the database and performed the statistical analysis. C-HC and S-HL wrote the first draft of the manuscript. C-HC, S-HL, and C-YW wrote sections of the manuscript. All authors contributed to the manuscript revision, and read and approved the submitted version.

## Conflict of Interest

The authors declare that the research was conducted in the absence of any commercial or financial relationships that could be construed as a potential conflict of interest.
